# Reliability and variation in mitochondrial respiration in human adipose tissue

**DOI:** 10.1080/21623945.2021.1991617

**Published:** 2021-11-24

**Authors:** Ronni Eg Sahl, Eva Frederikke Høy Helms, Malte Schmücker, Mathias Flensted-Jensen, Arthur Ingersen, Thomas Morville, Flemming Dela, Jørn Wulff Helge, Steen Larsen

**Affiliations:** a Xlab, Center for Healthy Aging – Department of Biomedical Sciences, University of Copenhagen, Copenhagen, Denmark; b Novo Nordisk Foundation Center for Basic Metabolic Research, Faculty of Health and Medical Sciences, University of Copenhagen, Copenhagen, Denmark; c Department of Geriatrics, Bispebjerg University Hospital, Copenhagen, Denmark; d Department of Clinical Research Centre, Medical University of Bialystok, Bialystok, Poland; e Mærsk Tower, Panum, Copenhagen-N, Denmark

**Keywords:** Adipose tissue, human, mitochondria, high resolution respirometry, coefficient of variation

## Abstract

Adipose tissue mitochondrial function is gaining increasing interest since it is a good marker of overall health. Methodological challenges and variability in assessing mitochondrial respiration in fresh adipose tissue with high-resolution respirometry are unknown and should be explored. Mitochondrial respiratory capacity (MRC) in human adipose tissue declines in a gradual manner when analyses are postponed 3 h and 24 h, with a statistically significant decline 24 h after obtaining the biopsy. This decline in MRC is associated with a reduced integrity of the outer mitochondrial membrane at both time points. This study suggests that the optimal amount of tissue to be used is 20 mg and that different technicians handling the biopsy do not affect MRC.

## Introduction

Mitochondrial function in human subcutaneous adipose tissue (AT) is closely linked to both ageing and disease [1–3] and is accepted as a good marker of overall health in the literature [4]. An interaction between mitochondrial dysfunction and inflammation in AT has been found [5], and inflammation leads to decreased insulin sensitivity and metabolic diseases [6,7]. A growing pile of research on the association between AT inflammation and mitochondrial function has accumulated over the last two decades (120-fold increase in publications in 20 years [8]). Thus, measurement of AT mitochondrial function can be used as an early marker of lifestyle diseases.

Most of the research on mitochondrial function has focussed on mitochondrial biomarkers (enzyme activity, protein content and mRNA expression), while the first article on mitochondrial respiratory capacity (MRC) using high-resolution respirometry, an ex vivo, physiology-mimicking method was first published in 2010 [9]. This method enables measurements of oxygen consumption when stimulating different respiratory complexes and the entire electron transport system (ETS) in intact mitochondria situated in an intact intra-cell organelle structure. Despite the advantages of this method and a large number of studies using this method during the last decade [10], we have not been able to find any evidence of the reliability and variation of this method in human white AT. We identified three relevant parameters of variation within this method. The parameters were investigated separately: 1) the effect of timing (postponing the analysis from 0 to 3 and 24 hours after the biopsy), 2) the effect of amount of tissue in the chamber (10 mg, 20 mg or 40 mg), and 3) the effect of different experienced technicians dissecting the biopsy. We also established the variation of the methodology and data analysis at different respiratory states.

## Methods and materials

### Test subjects

We recruited 17 healthy men (n = 10) and women (n = 7) (age 34 ± 12 years, BMI 23.1 ± 2.0 kg/m^2^). All subjects reported to our laboratory in the morning after an overnight fast and had abstained from strenuous exercise 24 hours before. They all gave written informed consent after thorough information about the study and risks involved. All procedures were carried out in accordance with the Declaration of Helsinki. The study was approved by the local Ethics Committee of Copenhagen and Frederiksberg in Denmark (H-18057641).

### Design

**Figure 1.**
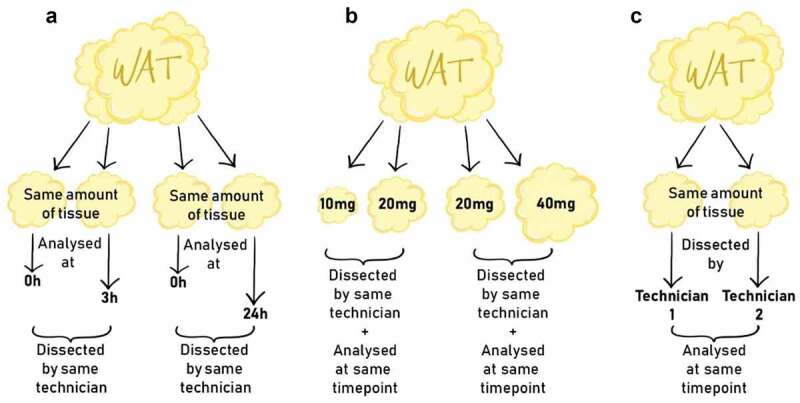
Experimental outline. (a) Effect of timing: Mitochondrial respiratory capacity (MRC) in the same amount of adipose tissue, dissected by the same technician and analysed at time point 0 hours (0 h) and postponed either 3 hours (3 h) or 24 hours (24 h), respectively. (b) Effect of amount of tissue used: MRC in 10 mg, 20 mg and 40 mg adipose tissue, dissected by the same technician and analysed at the same time point. (c) Effect of technician handling the biopsy: MRC in the same amount of adipose tissue, dissected by two different technicians and analysed at the same time point. Abbreviation: WAT; white adipose tissue

--We investigated the effect of timing, amount of tissue and the technician handling the biopsy when analysing fresh human subcutaneous AT using high-resolution respirometry. We changed and investigated one factor at a time. For the two factors, timing and amount, there were three different levels to investigate. Due to sample limitation, we did not have a full dataset with all three levels for all test subjects; thus, we compared them in pairs, using time point 0 h as benchmark in timing and 20 mg as benchmark in amount (see Figure 1). To investigate the impact of timing, we postponed dissection and measurement, and measured mitochondrial respiratory capacity (MRC) in fresh human AT from the same biopsy immediately after sampling (0 h), and either 3 hours (3 h) or 24 hours (24 h) after sampling. The biopsy was kept in BIOPS buffer (see specifications below) in a glass container (20 ml) with air tight sealing at 0 degrees Celsius until dissection. Samples were dissected by the same technician and the same amount of tissue was used.

To investigate the impact of different amounts of tissues, we measured MRC in either 10 mg versus 20 mg or 20 mg versus 40 mg of human AT from the same biopsy. Samples that were compared were dissected by the same technician at the same time point.

To investigate the impact of dissection by different technicians, we measured MRC in human AT from the same biopsy dissected by the same two technicians. Samples that were compared were measured in the same amount of tissue and at the same time point.

### Biopsy routine and preparation of sample

After 15 minutes of supine rest a biopsy was obtained from the subcutaneous AT from either the abdominal (n = 13) or gluteal (n = 4) area depots. The biopsy was obtained under local anaesthesia (Xylocaine, 20 mg/ml, (Aspen Pharmacare, Australia)) through a 10 mm incision using the Bergström needle technique with suction [11]. The AT was quickly dissected free of blood and connective tissue and placed in ice cold relaxing buffer (BIOPS (final concentrations): CaK_2_EGTA (2.77 mM), K_2_EGTA (7.23 mM), Na_2_ATP (5.77 mM), MgCl_2_ · 6 H_2_O (6.56 mM), taurine (20 mM), Na_2_Phospho-creatine (15 mM), Imidazole (20 mM), Dithiothreitol (0.5 mM), MES (50 mM), pH = 7.1, 0°C).

Under a magnifier the biopsy was dissected into smaller pieces using two small forceps, still being soaked in ice-cold BIOPS buffer. After dissection, all ATs were transferred into a glass vial with 5 ml of MIR05 buffer (final concentrations), sucrose (110 mM), potassium lactobionate (60 mM), EGTA (0.5 mM), MgCl_2_ · 6 H_2_O (3 mM), taurine (20 mM), KH_2_PO_4_ (10 mM), HEPES (20 mM), BSA (1 g/l), pH 7.1, 37°C) and washed for 10 minutes. After washing, the AT was quickly dried on soaking paper and then weighed and placed in an oxygraph chamber (Oxygraph-2K, Oroboros instruments, Innsbruck, Austria) containing MIR05 buffer at 37°C. All measurements were performed in duplicates. Data was recorded and analysed in DatLab software (Oroboros Instruments, Innsbruck, Austria).

### Mitochondrial respiration protocol

Immediately after the AT was placed in the oxygraph, the chamber was closed and digitonin (2 μM, final concentration) was added to permeabilize the adipocyte plasma membrane. Malate (2 mM), pyruvate (5 mM) and glutamate (10 mM) was added to measure state 2 respiration (LEAK), then ADP (5 mM) was added to measure state 3 respiration with complex I linked substrates (CI_p_). Succinate (10 mM) was added to measure complex I+II-linked respiration (CI+II_p_), followed by cytochrome c (10 µM) to test the integrity of the outer mitochondrial membrane. Oligomycin (2 µg/ml) was added to inhibit the ATP synthase (state 4o) and finally carbonyl cyanide-4-(trifluoromethoxy) phenylhydrazone (FCCP) was titrated in steps of 0.5 µM to test the capacity of the electron transport system (ETS). The total length of the protocol was 90–120 minutes.

### Statistics

All data were analysed in GraphPad Prism version 8.4.3 (GraphPad Software, La Jolla California, USA) assuming normal distribution and equal variance. A two-way ANOVA with repeated measures was used to analyse for differences in dissection, timing and amount. A paired t-test was used to analyse for differences in cytochrome *c* response. Coefficient of Variation (CV) was calculated as the mean divided by the standard deviation and expressed as percentage. CV was calculated for each duplicate measurement, and then all CVs were used to calculate the overall CV. Likewise, CV was calculated for each single measurement when two different researchers analysed the DatLab traces and the average of all these CVs was used to express the overall CV. Data is presented as mean ± SEM in the text and as boxplots with median and 95% CI in the figures.

We aimed at a sample size of nine in each group; however, some measurements were excluded in the data analysis phase, and we ended up with six to nine samples in each group.

## Results

### Effect of timing

**Figure 2.**
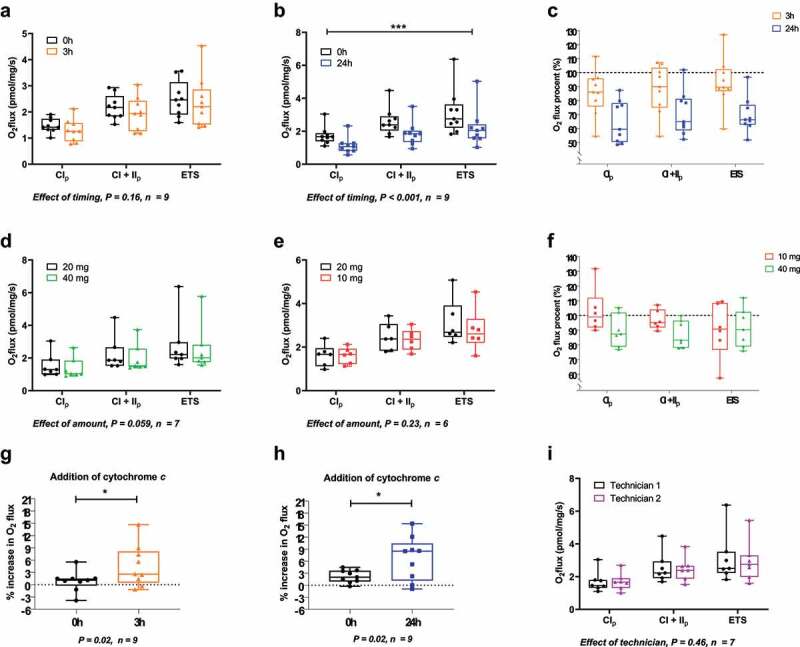
(a, b) Effect of postponed measurement (3 h and 24 h, respectively) in adipose tissue on complex I linked respiration (CI_p_), complex I+II linked respiration (CI+II_p_) electron transport system (ETS). (c) Complex I linked respiration (CI_p_), complex I+II linked respiration (CI+II_p_) and electron transport system (ETS) of all three time points (0 h, 3 h and 24 h) indexed to 0 h. (d, e) Effect of amount of adipose tissue on complex I linked respiration (CI_p_), complex I+II linked respiration (CI+II_p_) and electron transport system (ETS). (f): Complex I linked respiration (CI_p_), complex I+II linked respiration (CI+II_p_) and electron transport system (ETS) of all three amounts of adipose tissue (10, 20 and 40 mg) indexed to 20 mg. (g, h) Increase in respiration from complex I+II linked respiration (CI+II_p_) after addition of cytochrome c, when measurement is postponed 3 h and 24 h, respectively. (i): Effect of different technicians dissecting the biopsy on complex I linked respiration (CI_p_), complex I+II linked respiration (CI+II_p_) and electron transport system (ETS). (a, b, d, e, i) is analysed using a two-way ANOVA RM. (g) and (h) is analysed using paired t-test. All data shown as boxplots with medians and 95% confidence intervals. No statistics is performed on (c) and (f)

MRC was not statistically different between time point 0 h and 3 h (CI_p_: 1.5 ± 0.1 vs. 1.3 ± 0.1, CI+II_p_: 2.2 ± 0.2 vs. 1.9 ± 0.2 pmol, ETS: 2.5 ± 0.2 vs. 2.4 ± 0.3 pmol O_2_/mg/s for 0 h and 3 h, respectively, P = 0.16, Figure 2(a)). However, MRC was decreased (P < 0.001) at 24 h compared with 0 h (CI_p_: 1.7 ± 0.2 vs. 1.1 ± 0.2, CI+II_p_: 2.6 ± 0.3 vs. 1.9 ± 0.2, ETS: 3.2 ± 0.4 vs. 2.2 ± 0.4 pmol O_2_/mg/s for 0 h and 24 h, respectively, Figure 2(b)). In Figure 2(c) time points 3 h and 24 h are shown together, and data from **2A** and **2B** are indexed to time point 0 h.

MRC after addition of cytochrome *c* showed a significant increase from 0 h to 3 h (0.9% to 4.6%, P = 0.02, Figure 2(g)) and from 0 h to 24 h (2.2% to 6.7%, P = 0.02, Figure 2(h)).

### Effect of amount

We observed a non-significant tendency towards favouring 20 mg over 40 mg (CI_p_: 1.5 ± 0.3 vs. 1.4 ± 0.2, CI+II_p_: 2.2 ± 0.4 vs. 2.0 ± 0.3, ETS: 2.8 ± 0.6 vs. 2.6 ± 0.5 pmol O_2_/mg/s for 20 mg and 40 mg, respectively, P = 0.059, Figure 2(d), while we did not observe any difference between 10 mg and 20 mg (CI_p_: 1.6 ± 0.1 vs. 1.6 ± 0.2, CI+II_p_: 2.3 ± 0.2 vs. 2.5 ± 0.2, ETS: 2.8 ± 0.4 vs. 3.1 ± 0.4 pmol O_2_/mg/s for 10 mg and 20 mg, respectively, P = 0.23, Figure 2(e). In Figure 2(f) 10 mg and 40 mg are shown together, data from **2D** and **2E** are indexed to 20 mg.

We observed no difference in MRC after addition of cytochrome *c* for neither 20 mg vs. 40 mg (1.1% vs. 2.1%, P = 0.30, data not shown) nor 10 mg vs. 20 mg (0.7% vs. 2.9%, P = 0.37, data not shown).

### Effect of technician

We observed no difference in MRC when two different technicians dissected the biopsy (CI_p_: 1.7 ± 0.2 vs. 1.7 ± 0.2, CI+II_p_: 2.6 ± 0.3 vs. 2.5 ± 0.3 p, ETS: 3.1 ± 0.5 vs. 2.9 ± 0.4 pmol O_2_/mg/s for technician 1 and 2, respectively, P = 0.46, Figure 2(i)).

We observed no difference in MRC after addition of cytochrome *c* between technician 1 and 2 (2.0% vs. 0.6%, P = 0.21, data not shown).

### Duplicate CV

**Figure 3.**
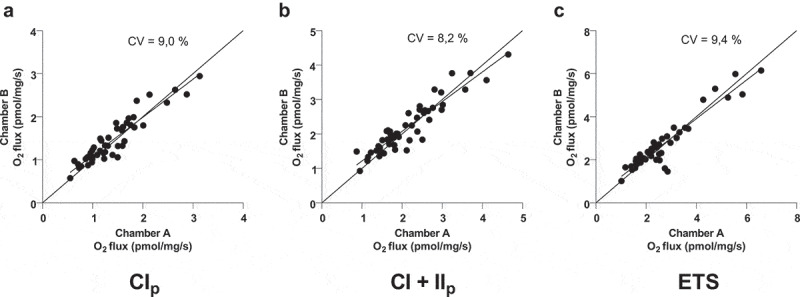
Each dot represents a duplicate measure of complex I linked respiration (CI_p_) (a), complex I+II linked respiration (CI+II_p_) (b) and electron transport system (ETS) (c). The coefficient of variation (CV) is calculated for each of the respiratory states. CV, line of identity and a simple linear regression line is shown in all three plots. n = 50 for all three plots

We calculated the overall coefficient of variation (CV) for complex I, complex I+II and ETS respiration states between the duplicate measurements for all three parameters (CV: 9.0%, 8.2% and 9.4%, respectively, n = 50, Figure 3).

### Difference between DatLab-trace analyses by researcher 1 and 2

**Figure 4.**
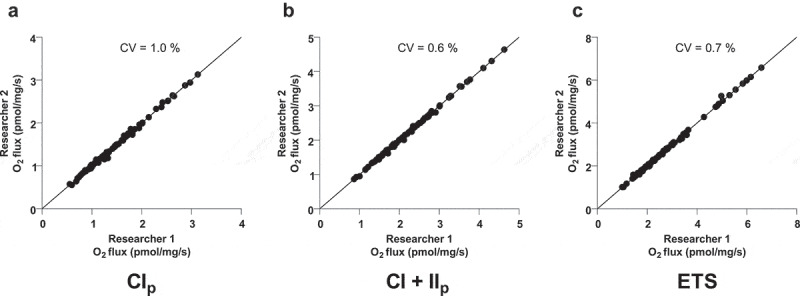
Each dot represents a single measurement performed in Datlab by researcher 1 and 2. MRC is shown for complex I linked respiration (CI_p_) (a), complex I+II linked respiration (CI+II_p_) (b) and electron transport system (ETS) (c). The coefficient of variation (CV) is calculated for each of the respiratory states. CV, line of identity and a simple linear regression line is shown in all three plots. n = 100 for all three plots

When two independent experienced researchers analysed each single measurement in DatLab, we found an overall CV in complex I, complex I+II and ETS respiration state of 1.0%, 0.6% and 0.7%, respectively (n = 100), Figure 4.

## Discussion

In this study we investigated the methodological variation of MRC in AT and how practical aspects, timing, tissue amount, biopsy handling and trace-analysis, can affect the measurements.

We demonstrated that AT MRC is lowered when analyses are postponed 24 hours (Figure 2(e)). Short time postponement of measurments is often necessary due to practical issues and we found no effect on complex I linked respiration (CI_p_), complex I+II linked respiration (CI+II_p_) and in the electron transport system (ETS) with 3 hours postponement (Figure 2(d). However, a numerical non-significant decrease (14%, 12% and 7% for CI_p,_ CI+II_p_ and ETS, respectively, *NS*) was observed which could indicate that a gradual decline in MRC is associated with postponement of measurements (Figure 2(f). It has been shown that MRC in skeletal muscle is not affected after 5 hours postponement, but that specific study did not investigate the possible effect of a long term postponement [12]. In 2002 Kuznetsov and colleagues showed that MRC in liver tissue decline when measurements are postponed [13]. Similar to our study there was a somewhat linear decline in MRC, becoming significant between 45–84 hours after biopsy sampling in the different respiratory states [13]. For both 3 and 24 hours postponement we observed an increased respiration after addition of cytochrome *c* (Figure 2(g,h)), which indicates that the integrity of the outer mitochondrial membrane is affected and therefore a possible contributor to a lower MRC. Whether this is due to the composition of the buffer, the storage temperature or other factors are currently unknown. We emphasize that any postponement of experiments with fresh human AT is minimized and that the same delay is used before and after an intervention.

We observed a tendency towards higher MRC in 20 mg compared to 40 mg of AT. A large amount of tissue means a faster decrease in oxygen concentration in the closed oxygraph chamber, and given the fact that human tissue is a limited source, the lowest amount of tissue needed, without compromising the analysis, is desirable. Since the use of 10 mg did not show any differences to 20 mg, it could be argued that this is a sufficient amount of tissue to be used. However, we did see more unstable traces of the O_2_ flux, which would complicate the data analysis process. We therefore consider the optimal amount to be approximately 20 mg.

No other study has thoroughly investigated the optimal amount of AT used with this method before. A study from 2010 in morbidly obese patients used 50 mg of AT, after pilot studies using between 25 and 300 mg [9]. The optimal amount of tissue is clearly subject to change with sex, age, body fatness, training status, AT depot, etc. In this study we used biopsies from both the gluteal and abdominal depots from healthy young to middle-aged females and males. Despite this rather broad range of criteria, the results should be interpreted with that in mind, and the optimal amount could be different in another setting.

We demonstrated that two experienced technicians dissecting the biopsies did not affect measurements (Figure 2(i)). Data from a previous study indicate that this is also the case with human skeletal muscle, when dissected by two skilled technicians [12]. The two technicians in our study adhered to the same procedure and used the same equipment in order to minimize variation.

We found a CV of 8.2% for complex I + II linked respiration in fresh AT (Figure 3(b)) which is in line with a previous study in fresh skeletal muscle, with a CV of 8.4% at the same respiration state [14]. We are pleased to find the variations to be similar, despite the very different morphology and function of skeletal muscle and AT. As demonstrated in the aforementioned study, most of the variation when measuring muscle tissue could be accounted for by the background variance of the method itself and not the heterogeneous nature of the muscle tissue. The reasonable low variation found in this study is therefore satisfying. Furthermore, we found a very low variation (CV: 0.6–1%) when two researchers analysed the Datlab traces independently, despite the fact that these analyses seem rather subjective, we do not see any indication that a human bias is introduced in the analysis part.

In summary, we found the methodology to have an acceptable variation. We highlight that storage time can affect measurements and the optimal amount of tissue used from healthy normal weight subjects seem to be approximately 20 mg. We did not find any evidence that different technicians handling biopsies affect measurements when they adhere to the same procedure. We emphasize that the same timing and amount should be used before and after an intervention.

## Data Availability

Data availability statement The data that support the findings of this study are at doi:10.17632/t2mp4f5fcw.1

## References

[1] Boengler K, Kosiol M, Mayr M, et al. Mitochondria and ageing: role in heart, skeletal muscle and adipose tissue. J Cachexia Sarcopenia Muscle. 2017;8(3):349–369. 28432755 10.1002/jcsm.12178 PMC5476857

[2] Jang JY, Blum A, Liu J, et al. The role of mitochondria in aging find the latest version : the role of mitochondria in aging. J Clin Invest. 2018;128(9):3662–3670. Available from: https://dm5migu4zj3pb.cloudfront.net/manuscripts/120000/120842/cache/120842.3-20180821165419-covered-253bed37ca4c1ab43d105aefdf7b5536.pdf 30059016 10.1172/JCI120842 PMC6118639

[3] Lee JH, Park A, and Oh KJ, et al. The role of adipose tissue mitochondria: regulation of mitochondrial function for the treatment of metabolic diseases. Int J Mol Sci. 2019;20(19: 4924). 10.3390/ijms20194924 PMC6801758 31590292

[4] Stephenson EJ, Hawley JA. Mitochondrial function in metabolic health: a genetic and environmental tug of war. Biochim Biophys Acta. 2014;1840(4):1285–1294. 24345456 10.1016/j.bbagen.2013.12.004

[5] Woo C-Y, Jang JE, Lee SE, et al. Mitochondrial dysfunction in adipocytes as a primary cause of adipose tissue inflammation. Diabetes Metab J. 2019;43(3):247–256. 30968618 10.4093/dmj.2018.0221 PMC6581541

[6] Bódis K, Roden M. Energy metabolism of white adipose tissue and insulin resistance in humans. Eur J Clin Invest. 2018;48(11):e13017. 30107041 10.1111/eci.13017

[7] Boudina S, Graham TE. Mitochondrial function/dysfunction in white adipose tissue. Exp Physiol. 2014;99(9):1168–1178. 25128326 10.1113/expphysiol.2014.081414

[8] Pubmed. Internet content. 2021. Available from: https://pubmed.ncbi.nlm.nih.gov/?term=adiposetissueinflammationmitochondrialfunction&sort=date&ac=no

[9] Kraunsøe R, Boushel R, Hansen CN, et al. Mitochondrial respiration in subcutaneous and visceral adipose tissue from patients with morbid obesity. J Physiol. 2010;588(12):2023–2032. 20421291 10.1113/jphysiol.2009.184754 PMC2911209

[10] OROBOROS. Internet content. 2021. Available from: https://wiki.oroboros.at/index.php/O2k-Publications:_Fat

[11] Shanely RA, Zwetsloot KA, Triplett NT, et al. Human skeletal muscle biopsy procedures using the modified Bergstrom technique. J Visualized Exp. 2014;(91):1–8. Available from: http://www.ncbi.nlm.nih.gov/pubmed/25285722 10.3791/51812 PMC4828068 25285722

[12] Cardinale DA, Gejl KD, Ørtenblad N, et al. Reliability of maximal mitochondrial oxidative phosphorylation in permeabilized fibers from the vastus lateralis employing high-resolution respirometry. Physiol Rep. 2018;6(4):1–8. 10.14814/phy2.13611 PMC5820461 29464938

[13] Kuznetsov AV, Strobl D, Ruttmann E, et al. Evaluation of mitochondrial respiratory function in small biopsies of liver. Anal Biochem. 2002;305(2):186–194. 12054447 10.1006/abio.2002.5658

[14] Sahl RE, Morville T, Kraunsøe R, et al. Variation in mitochondrial respiratory capacity and myosin heavy chain composition in repeated muscle biopsies. Anal Biochem. 2018 June;556:119–124. 29966588 10.1016/j.ab.2018.06.029

